# Treatment of focal degenerative cartilage defects with polymer-based autologous chondrocyte grafts: four-year clinical results

**DOI:** 10.1186/ar2638

**Published:** 2009-03-05

**Authors:** Peter C Kreuz, Sebastian Müller, Christian Ossendorf, Christian Kaps, Christoph Erggelet

**Affiliations:** 1 Department of Orthopaedic and Trauma Surgery, University Medical Center Rechts der Isar of the Technical University Munich, Ismaninger Str. 22, 81675 Munich, Germany; 2 Department of Orthopaedic and Trauma Surgery, University Medical Center Freiburg, Hugstetter Str. 55, 79106 Freiburg, Germany; 3 Department of Rheumatology, Charité Campus Mitte, Charité – Universitätsmedizin Berlin, Charitéplatz 1, 10117 Berlin, Germany

## Abstract

**Introduction:**

Second-generation autologous chondrocyte implantation with scaffolds stabilizing the grafts is a clinically effective procedure for cartilage repair. In this ongoing prospective observational case report study, we evaluated the effectiveness of BioSeed^®^-C, a cell-based cartilage graft based on autologous chondrocytes embedded in fibrin and a stable resorbable polymer scaffold, for the treatment of clinical symptomatic focal degenerative defects of the knee.

**Methods:**

Clinical outcome after 4-year clinical follow-up was assessed in 19 patients with preoperatively radiologically confirmed osteoarthritis and a Kellgren-Lawrence score of 2 or more. Clinical scoring was performed before implantation of the graft and 6, 12, and 48 months after implantation using the Lysholm score, the Knee injury and Osteoarthritis Outcome Score (KOOS), the International Knee Documentation Committee (IKDC) score, and the International Cartilage Repair Society (ICRS) score. Cartilage regeneration and articular resurfacing were assessed by magnetic resonance imaging (MRI) 4 years after implantation of the autologous cartilage graft.

**Results:**

Significant improvement (*P *< 0.05) of the Lysholm and ICRS scores was observed as early as 6 months after implantation of BioSeed^®^-C and remained stable during follow-up. The IKDC score showed significant improvement compared with the preoperative situation at 12 and 48 months (*P *< 0.05). The KOOS showed significant improvement in the subclasses pain, activities of daily living, and knee-related quality of life 6 months as well as 1 and 4 years after implantation of BioSeed^®^-C in osteoarthritic defects (*P *< 0.05). MRI analysis showed moderate to complete defect filling with a normal to incidentally hyperintense signal in 16 out of 19 patients treated with BioSeed^®^-C. Two patients without improvement in the clinical and MRI scores received a total knee endoprosthesis after 4 years.

**Conclusions:**

The results show that the good clinical outcome achieved 1 year after implantation of BioSeed^®^-C remains stable over the course of a period of 4 years and suggest that implanting BioSeed^®^-C is a promising treatment option for the repair of focal degenerative defects of the knee.

## Introduction

Cartilage lesions of the knee occur frequently and represent a major health problem. Consecutive knee arthroscopies showed that up to 63% of the patients with knee-related symptoms suffered from chondral or osteochondral defects [1,2]. These defects comprise focal osteochondral or chondral lesions in 67%, osteoarthritic defects in 29%, lesions related to osteochondritis dissecans in 2%, and other defects in 1% of the cases [3]. Recently, a variety of surgical techniques that aim for resurfacing and regenerating of the articular cartilage have evolved. In the clinical routine, debridement, bone marrow-stimulating techniques, osteochondral autograft transfer, and autologous chondrocyte implantation (ACI) are commonly used cartilage repair techniques [4-8].

The first ACI was performed in 1987, and the clinical study of Brittberg and colleagues [4] in 1994 represents the starting point of cell-based cartilage repair and regenerative medicine. Up to now, more than 15,000 patients worldwide have been treated with ACI [9], and various reports documented the clinical effectiveness of implanting autologous culture-expanded chondrocytes for cartilage repair [10-13]. Although there is no significant evidence that ACI produces superior clinical results for the treatment of full-thickness articular cartilage defects compared with other cartilage repair interventions [14,15], it is regarded as a second-line treatment for small defects and a first-line treatment for defects larger than 2 to 4 cm^2 ^[16].

For ACI, a small partial or full-thickness cartilage biopsy is taken from a less weight-bearing area of the healthy articular cartilage. The chondrocytes are harvested by enzymatic digestion and cells are grown with autologous serum. For chondrocyte implantation, a periosteal flap or a collagen sheet is sutured to the surrounding healthy cartilage rim, creating a reservoir for the injection of the autologous chondrocyte cell suspension. The need for an intact cartilage rim limits the use of ACI to some regions of the knee, and the covering of the chondrocyte suspension with a periosteal flap or a collagen sheet may be insecure (for instance, in degenerative defects that often miss an intact cartilage rim). In addition, potential sources of complications may include periosteal hypertrophy, loosening of the periosteal flap, ablation, and loss of cells into the joint cavity [17-19]. These technical disadvantages of ACI result in re-operations in up to 25% to 36% of the patients [20,21]. Therefore, cartilage tissue engineering grafts that address these disadvantages by using three-dimensional scaffolds stabilizing the graft and the regenerative potential of autologous chondrocytes were developed. Meanwhile, clinical results have shown the effectiveness of hyaluronan-based [22,23], collagen-based [24,25], and resorbable polymer-based [26] autologous chondrocyte grafts for the repair of cartilage defects.

Currently, ACI is contraindicated in osteoarthritic patients. Nevertheless, preclinical studies suggest that chondrocytes or mesenchymal stem cells from osteoarthritic patients may have the capacity to form cartilage repair tissue and fulfill the prerequisites for use in ACI [27,28]. However, for cell-based cartilage therapies in osteoarthritis, it is important to harvest unaffected healthy cartilage biopsies since healthy chondrocytes have been shown to form a cartilage tissue *in vitro *that shows better morphology and a higher proteoglycan content than chondrocytes derived from osteoarthritic joints [29]. Clinically, it has been shown that microfracture treatment of patients with moderate osteoarthritis improved their pain and activity of daily living and significantly widened the joint spaces 1 year after treatment compared with the preoperative situation [30]. The effectiveness of a second-generation cartilage graft based on hyaluronan has been shown for the treatment of osteoarthritic knees with osteoarthritis not inhibiting the regeneration sequence [31].

Recently, it was shown that the autologous cartilage graft BioSeed^®^-C (BioTissue Technologies GmbH, Freiburg, Germany) based on a bioresorbable two-component gel-polymer scaffold is effective for the treatment of traumatic and focal osteoarthritic cartilage defects of the knee [26]. The aim of the present study was to evaluate ACI using BioSeed^®^-C for the treatment of mild degenerative and focal osteoarthritic defects of the knee. Magnetic resonance imaging (MRI) analysis of the cartilage repair tissue as well as the clinical evaluation of a series of 19 patients with pre-existing osteoarthritic symptoms and a 4-year clinical follow-up document the effectiveness of BioSeed^®^-C for the treatment of focal degenerative cartilage defects.

## Materials and methods

### Patients

From December 2001 to October 2002, 79 patients with traumatic and degenerative chondral defects of the knee joint were treated with a second-generation ACI (BioSeed^®^-C). Patients suffered from traumatic, mild degenerative, or osteoarthritic and symptomatic defects of the articular cartilage of the knee which were clinically significant [26]. The study was performed in compliance with the ethical review board of the University of Freiburg, Germany. All patients gave their consent to participate. Radiographs were taken preoperatively, and osteoarthritic degenerations were evaluated by two independent observers using the Kellgren-Lawrence scoring system. The observers were blinded to the procedure. A Kellgren-Lawrence score of greater than or equal to 2 defines osteoarthritis [32] and was found in 24 patients. Nineteen patients gave consent to a clinical follow-up of 4 years. Clinical examinations were performed at 0, 6, 12, and 48 to 60 months.

Characteristics of patients with degenerative cartilage defects are presented in Table 1. The average age of patients (8 females and 11 males; mean body mass index of 25, ranging from 19 to 34) was 35 years (25 to 50 years). The mean defect size of the first lesion was 4 cm^2 ^(2 to 6 cm^2^). All defects (first lesion) were classified as Outerbridge class IV [33] and were located on the medial femoral condyle (n = 14), the lateral femoral condyle (n = 2), or the patella (n = 3). Four patients had a second chondral defect that was treated with ACI as well. Previous surgical procedures were shaving (n = 10), abrasion arthroplasty (n = 4), drilling/microfracture (n = 1), meniscectomies (n = 6), anterior cruciate ligament/collateral ligament reconstructions (n = 7), or high tibial osteotomy (n = 5).

**Table 1:** Characteristics of patients with degenerative cartilage defects

Characteristic	Patient data
Gender	Female (n = 8), male (n = 11)
Age, years	35 (range 25–50)
Height, cm	173 (range 162–184)
Weight, kg	75 (range 57–100)
Body mass index	25 (range 19–34)
Kellgren-Lawrence score	2 (n = 10) 3 (n = 9)
Defect size of 1st lesion, cm^2^	4 (range 2–6)
Outerbridge classification of 1st lesion	IV (n = 19)
Localization of 1st lesion	Medial femoral condyle (n = 14) Lateral femoral condyle (n = 2) Patella (n = 3)
2nd lesion	n = 4
Defect size of 2nd lesion, cm^2^	3 (range 2–4)
Outerbridge classification of 2nd lesion	IV (n = 4)
Localization of 2nd lesion	Medial femoral condyle (n = 1) Trochlea (n = 3)
Concomitant surgeries	High tibial osteotomy (n = 5) Anterior cruciate ligament reconstruction (n = 4)
Previous surgical procedures	High tibial osteotomy (n = 5) Shaving (n = 10) Abrasion arthroplasty (n = 4), Microfracture/drilling (n = 1) Meniscectomy (n = 6) Anterior cruciate ligament/collateral ligament reconstruction (n = 7)

### Implantation of BioSeed^®^-C and follow-up treatment

**Figure 1:**
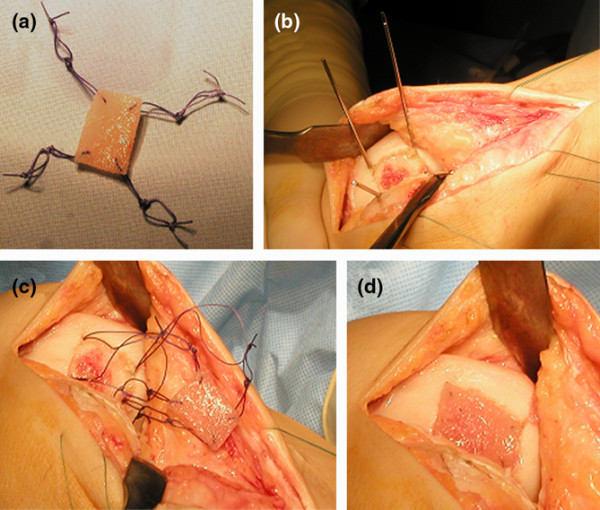
Arthrotomic implantation of BioSeed^®^-C. **(a) **BioSeed^®^-C was armed in each corner with resorbable threads secured by threefold knots. **(b) **In every corner of the defect, k-wires were drilled using the inside-out technique. **(c) **Guiding threads were pulled through the femoral bone using the k-wires, and the knots were guided into the subchondral bone. **(d) **The knots serve as anchors, seizing the subchondral bone and securely fixing the graft.

For preparation of BioSeed^®^-C, autologous chondrocytes were harvested from healthy cartilage (approximately 250 mg) of a less weight-bearing area of the knee. One hundred milliliters of whole blood was collected with a conventional blood-sampling system (Sarstedt AG, Nümbrecht, Germany) and used for autologous chondrocyte growth. Twenty million chondrocytes were rearranged in fibrin and a polymer-based scaffold (2 × 3 cm and 0.2 cm in height) of polyglycolic/polylactic acid (polyglactin, vicryl) and polydioxanone. After careful debridement of the defective cartilage down to the subchondral bone, the graft was fitted to the size of the defect and implanted arthrotomically. Fixation of the graft (Figure 1) was achieved by transosseous anchoring as described previously [34]. Starting the day after surgery, patients were subjected to continuous passive motion. For 6 weeks, partial loading with 15% of body weight as well as isometric tension exercises were allowed. In weeks 7 to 12, patients increased the loading and performed strengthening exercises and active physiotherapy at a gentle level. From week 13 on, patients gradually increased the weight and performed muscular and coordination exercises up to full weight-bearing. Gentle exertion was allowed after 6 months and more strenuous activities and contact sports after 12 months.

### Evaluation of clinical results

For evaluation of clinical results after implantation of BioSeed^®^-C, the Lysholm score [35], the Knee injury and Osteoarthritis Outcome Score (KOOS) [36], and the International Knee Documentation Committee (IKDC) Knee Examination Form [37] were applied. The Knee Examination Form (surgeons' part) of the International Cartilage Repair Society (ICRS) cartilage injury evaluation package evaluates the subgroups knee joint effusion, passive motion deficit, ligaments, compartment, harvest site pathology, joint space, and functionality [38]. The score was applied and grading was performed from 1 (normal) to 4 (severely abnormal). The lowest grade within a group determines the group rate, and the worst group grade determines the final evaluation. The clinical situation was documented before and 6, 12 as well as 48 to 60 months after implantation of the graft. Forty-eight to sixty months after transplantation, repair and resurfacing of cartilage defects (n = 17) were evaluated with a state-of-the-art 1.5 Tesla MRI scanner (Siemens AG, Erlangen, Germany) and the Henderson scoring system was applied [13]. Hypertrophic changes were classified with the Kreuz score [19].

### Statistical analysis

For statistical analysis of the Lysholm and ICRS scores, the Kruskal-Wallis test and the one-way analysis of variance (ANOVA) of ranks test were used. For isolating the groups that differed significantly (*P *< 0.05) from the others, the all-pairwise multiple comparison procedure (Dunn's method) was applied. Statistical analysis of the IKDC data was performed using ANOVA followed by the *t *test. Differences were considered significant at a *P *value of less than 0.05. For analysis of the KOOS, the non-parametric Mann-Whitney rank sum test was applied and differences were considered significant at a *P *value of less than 0.05. All comparisons were performed between scorings at every individual point in time of the follow-up period compared with the preoperative scores.

## Results

### Postoperative findings in patients with focal degenerative cartilage defects treated with BioSeed^®^-C

BioSeed^®^-C was implanted arthrotomically using a transosseous fixation technique (Figure 1). The degenerated cartilage was debrided down to the subchondral bone, and the graft was fitted to the size of the defect. For fixation, the graft was armed in each corner with resorbable threads forming loops that were secured by threefold knots (Figure 1a). On every corner of the defect, a k-wire with a thread guide was drilled using the inside-out technique (Figure 1b). The k-wires that carry guiding threads thread through the guide of the k-wire, and the loops of the graft were pulled (inside-out) through the femoral bone (Figure 1c). The threefold knots act as anchors that seize within the subchondral bone and thus securely fix the graft in the defect (Figure 1d).

**Figure 2:**
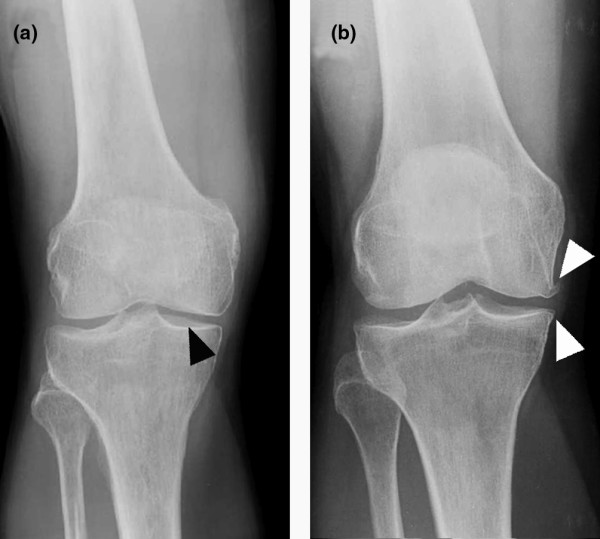
Radiographs of patients with focal degenerative cartilage defects prior to treatment with autologous chondrocyte grafts (BioSeed^®^-C). **(a) **This patient showed a Kellgren-Lawrence score of 2, with narrowing of the joint space (black arrowhead). **(b) **This patient showed a Kellgren-Lawrence score of 3, with narrowing of the joint space and osteophytes (white arrowheads).

BioSeed^®^-C was implanted in focal degenerative cartilage defects of knees that showed radiological signs of osteoarthritic degeneration (Figure 2). Applying the Kellgren-Lawrence score to the preoperatively performed radiographs showed that 24 patients had osteoarthritis with a Kellgren-Lawrence score of 2 with constriction of the joint space (Figure 2a, black arrowhead) or a score of 3 with constriction and formation of osteophytes (Figure 2b, white arrowheads). Nineteen out of twenty-four patients gave consent to clinical follow-up conducted 4 years after implantation of the graft. Postoperatively, no clinical signs of persistent knee joint infection or allergic reactions were evident. Knee joint effusion or swelling was reported by 9 patients. Symptoms of temporary blocking were observed in 4 out of 19 patients. None of the patients acquired potentially graft-related autoimmune disorders or signs of hypersensitivity. There were no signs of malignant transformation, migration of chondrocytes, poisoning, toxicity, organ failure, hepatic or renal disorders, or reproductive defects or teratogenic effects. There were no signs of loosening, debonding, or ablation of the graft. Minimal asymptomatic cartilage hypertrophic changes (<125%) were found in 3 patients, and abnormal cartilage growth was not evident. Nine patients were subjected to second-look arthroscopy due to symptoms like persistent grinding, catching, pain, or swelling. The newly formed repair tissue showed good integration and bonding as well as a visible contrast in color to the surrounding tissue. In 1 patient, multiple lesions scattered across the defect site and the retropatellar cartilage were observed. In 2 patients, a new cartilage lesion in the surroundings of the transplanted area was detected and treated with abrasion chondroplasty. In two other patients with persistent pain, the ACI procedure failed and a total knee endoprosthesis was implanted 4 years after implantation of the graft.

### Clinical evaluation of surgical results four years after implantation of BioSeed^®^-C

**Figure 3:**
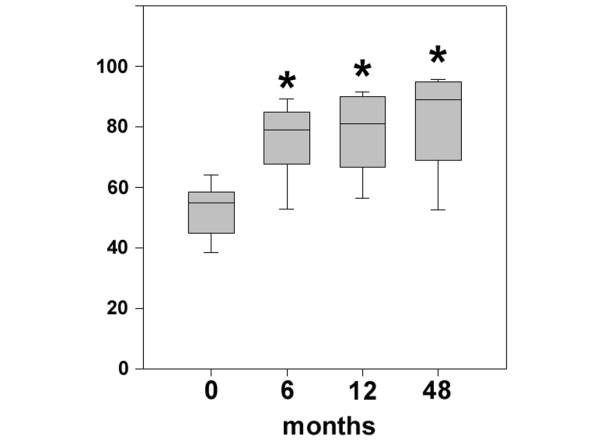
Clinical outcome after four years as evaluated by the Lysholm score. Statistical analysis of the clinical outcome as assessed by the Lysholm score was performed using analysis of variance on ranks (*P *< 0.00001) and subsequently the all-pairwise comparison according to Dunn's method. Scores are presented as the median, with the ends of the boxes defining the 25th and 75th percentiles and error bars defining the 10th and 90th percentiles. Where indicated (asterisks), differences were statistically significant (*P *< 0.05) compared with the preoperative situation.

**Figure 4:**
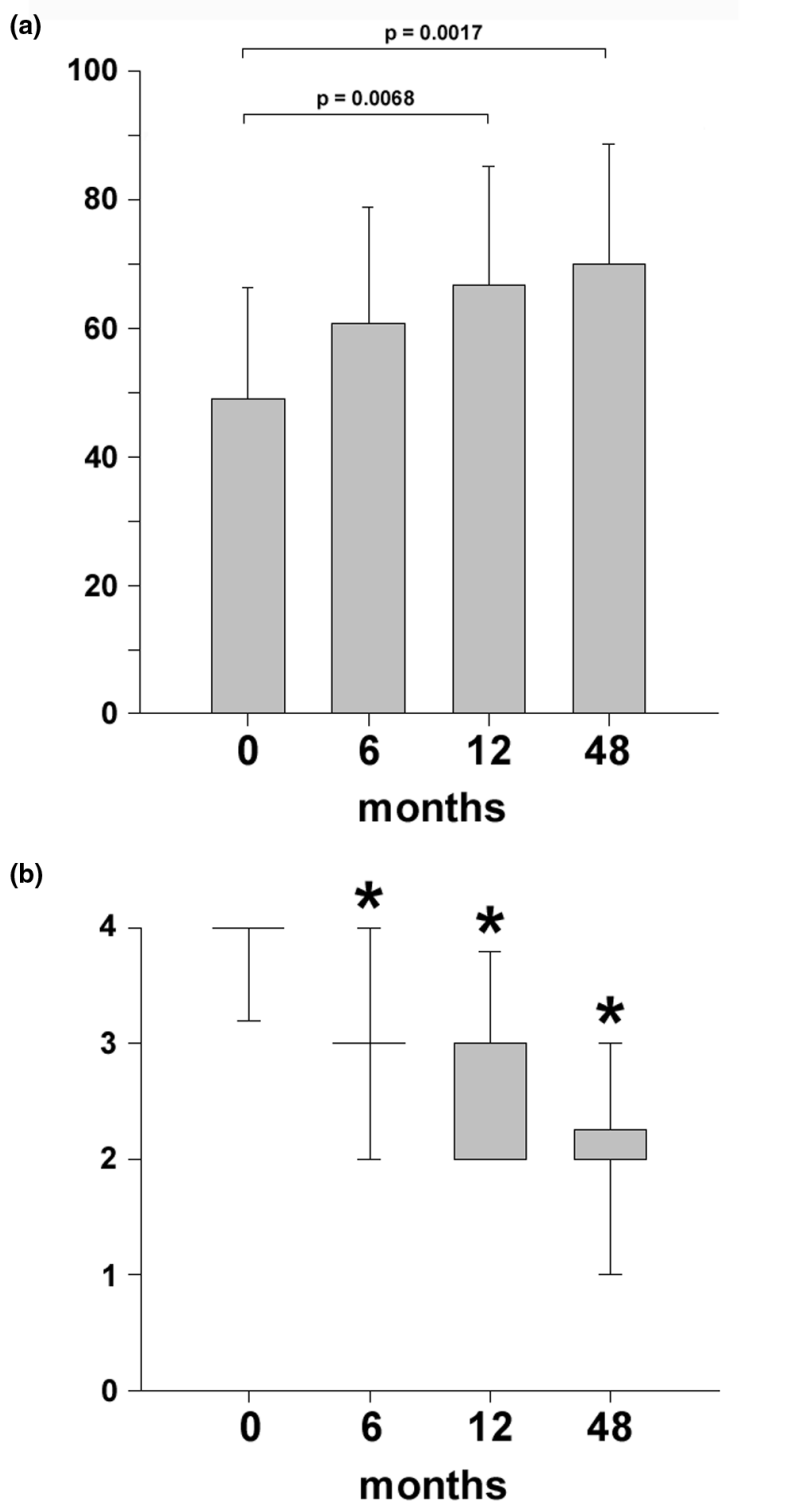
Clinical outcome after four years as evaluated by the International Knee Documentation Committee (IKDC) and International Cartilage Repair Society (ICRS) scores. **(a) **Statistical analysis of the clinical outcome as assessed by the IKDC subjective knee evaluation score was performed using analysis of variance (*P *< 0.007) and subsequently the *t *test. Scores are presented as the mean, with error bars defining standard deviation. **(b) **The ICRS scores were statistically analyzed by using analysis of variance on ranks (*P *< 0.000001) and subsequently the all-pairwise comparison according to Dunn's method. Scores are presented as the median, with the ends of the boxes defining the 25th and 75th percentiles and error bars defining the 10th and 90th percentiles. Where indicated (asterisks), differences were statistically significant (*P *< 0.05) compared with the preoperative situation.

As assessed by the Lysholm score, statistically significant improvements (*P *< 0.05) were observed as early as 6 months after implantation of BioSeed^®^-C (Figure 3). Compared with preoperative findings, the median Lysholm score significantly improved (*P *< 0.05), increasing from 55.0 to 89.0 in patients with focal osteoarthritic degeneration 4 years after implantation of the graft. The clinical outcome 4 years after implantation of BioSeed^®^-C in osteoarthritic focal defects was evaluated using the IKDC subjective knee evaluation score and the ICRS score (Figure 4). The IKDC score (Figure 4a) showed significant improvement 1 year (*P *= 0.0068) and 4 years (*P *= 0.0017) postoperatively compared with the preoperative situation. The mean score increased from 49.0 to 70.1 after 4 years. In addition, the ICRS score improved significantly (*P *< 0.05) over the whole study period from 4.0 preoperatively to 2.0 at 4-year follow-up (Figure 4b).

**Figure 5:**
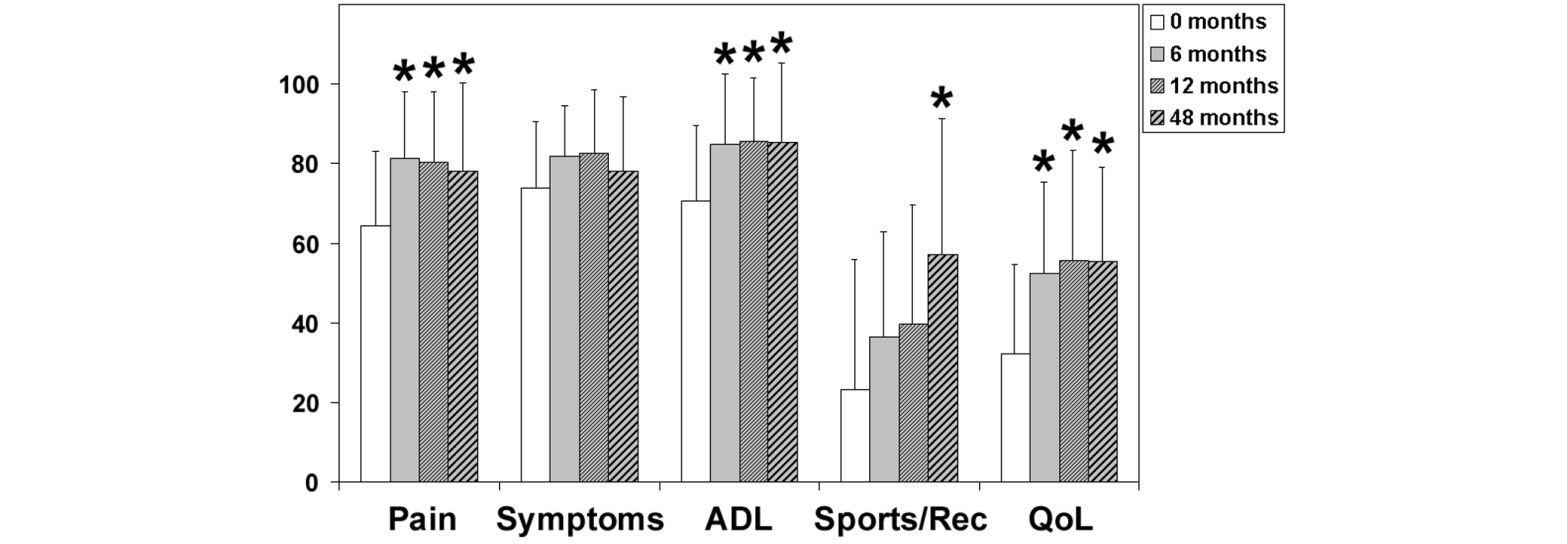
Clinical outcome after four years as evaluated by the Knee injury and Osteoarthritis Outcome Score (KOOS). The KOOS is presented as a mean value, and error bars define standard deviation. Where indicated (asterisks), differences were statistically significant (*P *< 0.05) compared with the preoperative situation as assessed by the Mann-Whitney rank sum test. ADL, activities of daily living; QoL, quality of life; Sports/Rec, sports and recreation.

The KOOS describes the patient's view about his knee and associated problems (Figure 5). At 6-month, 1-year, and 4-year follow-up, the patients' status improved significantly (*P *< 0.05) compared with preoperative findings. The median scores increased in the subclasses pain (69 to 89), activities of daily living (72 to 96), and knee-related quality of life (25 to 56) 4 years after implantation of the graft. Patients showed a significant improvement in the subclass sports and recreation (10 to 65) 4 years after implantation, whereas the subclass symptoms showed no significant improvement (*P *> 0.05) of the score (71 to 82). Both patients who needed total knee replacement after 4 years did not improve in the scores over the study period (*P *< 0.05). After 48 months, the Lysholm score was 38.5 (24 to 53) points and the KOOS subclasses symptoms and activity of daily life were 41 (38 to 44) points each and significantly worse compared with the results of the other patients (*P *< 0.05).

### Magnetic resonance imaging four years after transplantation of BioSeed^®^-C

**Figure 6:**
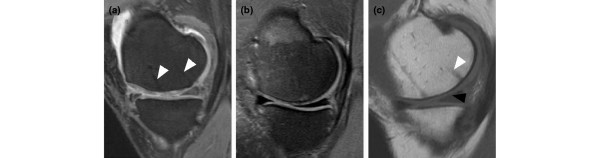
Magnetic resonance imaging (MRI) four years after implantation of BioSeed^®^-C. **(a) **Out of 17 patients, 11 patients, including this one, showed complete filling of the defect four years after implantation of BioSeed^®^-C. **(b) **Five patients, including this one, showed more than 50% defect filling but not complete defect filling. **(c) **One patient showed less than 50% defect filling (black arrowhead). The repair tissue gives a slightly altered MRI signal, and transosseous drill holes are still evident (white arrowheads).

**Figure 7:**
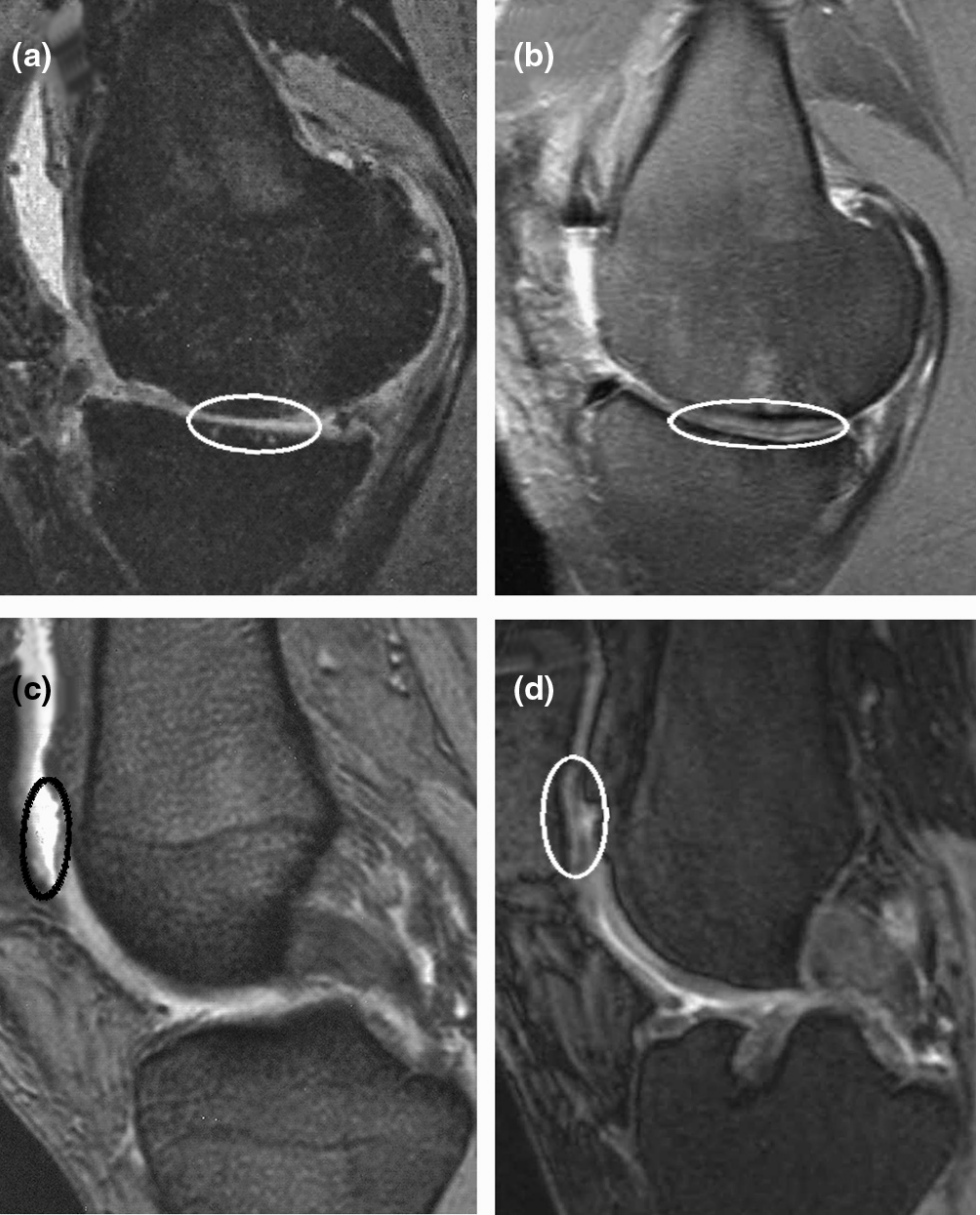
Magnetic resonance imaging (MRI) before and four years after implantation of BioSeed^®^-C. **(a) **Preoperative MRI shows a cartilage defect (encircled) at the medial femoral condyle. **(b) **After four years, MRI documented complete filling of the defect. Preoperatively, MRI shows a patellar **(c) **cartilage defect (encircled) that was completely filled after implantation of the graft as assessed by MRI at four years **(d)**.

Two patients had to undergo revision surgery and received a total knee endoprosthesis. Therefore, 17 out of 19 patients were analyzed by MRI 4 years after implantation of BioSeed^®^-C. Patients with degenerative cartilage defects treated with BioSeed^®^-C showed moderate to complete filling of the defects (Figure 6). Eleven patients showed a complete filling of the defect with cartilage repair tissue (Figure 6a). In 5 patients, the defects were filled more than 50% (Figure 6b), and 1 patient showed a defect fill of less than 50% (Figure 6c). The transosseous drill holes were still visible (Figure 6a,c, white arrowheads). Representative MRIs as assessed preoperatively and at 4-year follow-up (Figure 7) show that cartilage defects at the medial femoral condyle (Figure 7a,b) and patellar defects (Figure 7c,d) were completely filled with cartilage repair tissue after transplantation of the graft. A detailed MRI analysis according to Henderson and Kreuz is given in Table 2. The cartilage signal in 16 out of 17 defects was normal or showed slight alterations in the intensity. In one defect, the signal was hyperintense throughout large areas of the repair tissue. Strong to moderate subchondral edema was evident in 6 patients, and 11 out of 17 patients showed no or mild edema. Five patients showed moderate to strong signs of knee joint effusion. No to mild knee joint effusion was evident in 12 out of 17 patients treated with BioSeed^®^-C at 4-year follow-up. Both patients who sustained a total knee endoprosthesis after 4 years had the last control MRI after 12 months. The defect area was partially filled (<50%) with a hyperintensive repair tissue and showed a concomitant moderate subchondral edema.

**Table 2:** Magnetic resonance imaging analysis four years after transplantation of the graft in focal degenerative cartilage defects

Characteristic	Magnetic resonance imaging score			
	
	1 (normal)	2 (mild)	3 (moderate)	4 (abnormal)
Defect filling	11 (complete filling), including 3 with minimal hypertrophic changes (<125%)	5 (>50%)	1 (<50%)	0 (no filling)
Signal intensity	8 (normal)	8 (some hyperintense areas)	1 (hyperintense areas)	0 (no signal)
Subchondral edema	4 (no edema)	7 (mild edema)	5 (moderate edema)	1 (distinct edema)
Effusion	1 (no effusion)	11 (mild effusion)	4 (moderate effusion)	1 (distinct effusion)

## Discussion

The autologous gel-polymer cartilage graft, BioSeed^®^-C, uses the well-known regenerative capacity of autologous chondrocytes, gel-like matrices for promoting tissue formation, and the initial mechanical stability of resorbable polymer scaffolds for cartilage repair [39]. The assembly of chondrocytes in polymer-based scaffolds ensures the even distribution of a high number of vital chondrocytes within the graft and has been shown to allow the production of cartilage grafts that develop toward hyaline cartilage *in vitro *[40]. In particular, the embedding of culture-expanded chondrocytes in fibrin and polymer-based scaffolds made of polyglycolic acid or copolymers of polyglycolic and polylactic acid initiates chondrocyte re-differentiation *in vitro *and allows for formation of cartilage matrix *in vivo *after implantation of the graft [41,42]. The formation of cartilage repair tissue after implantation of the polymer-based graft made of non- and cryo-preserved chondrocytes has been shown in the rabbit joint defect model [43] as well as in a large animal horse model. In Haflinger horses, full-thickness cartilage defects of the fetlock joint were treated with the autologous polymer-based cartilage graft, and formation of a hyaline-like cartilage repair tissue as well as firm bonding of the graft to the adjacent healthy cartilage and to the subchondral bone tissue were evident 1 year after implantation [44]. Since the chondrocytes are embedded in and protected by the fibrin-polymer matrix, BioSeed^®^-C allows for easy handling of the graft during surgery, avoids the use of cover materials like periosteum or collagen sheets, needs no healthy cartilage rim surrounding the defect, and ensures arthroscopical implantation and secure fixation [34]. In particular, the secure fixation of cartilage grafts is of importance to avoid transplant loosening, debonding of the graft or ablation, and in turn clinical complications and re-operations. As assessed in recent biomechanical studies, stable second-generation cartilage grafts for ACI like BioSeed^®^-C allow good anchoring of the graft in the defect by fibrin gluing, chondral or transosseous suturing, and resorbable pin fixation [45-47]. From the cellular point of view, particularly in an osteoarthritic environment, joint homeostasis and the composition of the synovial fluid may be of special importance in cartilage repair. In a goat model, it has been shown that defects showed better repair when the lesion was covered immediately with periosteum compared with defects that were left untreated before transplantation [48]. Using a chick limb bud assay, synovial fluid from patients with acute traumatic cartilage defects has been shown to stimulate chondrogenesis, but synovial fluid from patients with chronic traumatic defects predominantly inhibited chondrogenic development [49]. In contrast, synovial fluid obtained from patients suffering from trauma, osteoarthritis, or inflammatory rheumatoid arthritis stimulated the synthesis of proteoglycans in a bovine model, with osteoarthritis and trauma synovial fluid showing a markedly increased synthesis compared with rheumatoid arthritis [50]. In a rabbit model, it has been shown that synovial fluid promotes and enhances cartilage tissue development from perichondrium [51]. Although the influence of synovial fluid or components of the fluid on cartilage repair remains unclear and further studies are needed, it is evident that an altered joint environment may influence tissue regeneration. Cartilage repair should be performed as early as possible and an inflammatory environment should be avoided.

From the clinical point of view, the use of autologous chondrocytes in suspension like in first-generation ACI has been shown to be effective for the repair of localized traumatic defects [10,11,52]. Second-generation cartilage grafts using scaffolds based on collagen or hyaluronan for stabilizing autologous chondrocytes are considered to be technically more attractive than first-generation ACI and have been shown to be clinically as effective as 'classical' ACI [22,24]. In a recent report of the treatment of traumatic and degenerative defects [26] and in this case series, we demonstrated the safety and effectiveness of the autologous cartilage gel-polymer graft BioSeed^®^-C for the treatment of challenging defects like large focal degenerative full-thickness cartilage lesions of the knee. After 1-year follow-up, mean scores increased significantly between 30% and 51% compared with the preoperative situation, depending on which score was analyzed. These good results lasted and showed significant improvement of the mean scores between 34% and 55% 4 years after implantation of the graft. This indicates a significant decrease in the patients' pain and knee instabilities during activity as well as a significant increase in patients' quality of life. The implantation of BioSeed^®^-C in focal osteoarthritic defects showed significant improvement in the KOOS subclasses pain, activity of daily living, and quality of life 1 year and 4 years after implantation of the graft. Obviously, the efficacy of the polymer-based autologous cartilage graft BioSeed^®^-C for the repair of osteoarthritic cartilage defects is shown by the improvement of clinical scores, by patients' pain and quality of life as well as by the good filling of the defects with repair tissue as assessed by MRI. This is of special relevance since the BioSeed^®^-C-mediated cartilage repair was achieved in a challenging patient cohort with clinically osteoarthritic symptoms and focal cartilage degeneration 1 and 4 years after implantation of the graft. Interestingly, the status of the patient 2 years after ACI is considered an important indicator for the future outcome. During this time, most of the complications of cell-based cartilage repair as well as improvement in clinical scores and subjective patient satisfaction were found. On the other hand, indicators of a worse outcome like multiple surgical procedures, higher age, and large defects correspond to findings published by others [24,53]. In general, for measuring and evaluating the clinical outcome of a given treatment strategy, patients' satisfaction and improvement are most important and are best assessed by well-established clinical outcome scores. From the scientific point of view, additional detailed questions arise regarding measurable parameters like the morphology and quality of the formed repair tissue, chondrocyte viability and distribution within the regenerative tissue as well as defect filling and graft integration. These issues can be addressed by non-invasive MRI techniques as shown here or by dGEMRIC (delayed gadolinium-enhanced MRI of cartilage) [54] as well as by minimally invasive histological evaluation [13,26,55]. These techniques may offer measurable insights in clinical outcome and value of the graft and may open avenues for developing precise indicators for the clinical outcome and prognosis. However, for a comprehensive evaluation of a regenerative cartilage repair therapy, both approaches are mandatory, giving clinical scores for patients' satisfaction and improvement as well as MRI and/or histological analysis for repair tissue evaluation.

After implantation of BioSeed^®^-C, 9 out of 19 patients were subjected to second-look arthroscopy due to symptoms like grinding, catching, pain, or swelling. This re-intervention rate, though for diagnostic purposes, is relatively high and may be related to this challenging patient cohort with a considerable number of concomitant surgeries like high tibial osteotomy and anterior cruciate ligament reconstruction. However, only 4 out of the 19 patients (21%) had to undergo revision surgery or re-operation. This is in concordance with other studies reporting rates of revision surgery of between 0% [56] and 25% [20]. Two patients were treated with an abrasion chondroplasty for newly formed cartilage lesions beside the transplanted area. Two additional patients out of the 19 patients received a total knee endoprosthesis and were classified as 'graft failure', although there is no evidence that the prosthesis was indicated because of a failure of BioSeed^®^-C. This failure rate of 10% corresponds to earlier findings describing rates of failure in ACI with other implants of between 5% [12] and 13% [20]. These patients with focal degenerative defects treated with the chondrocyte graft were young and in the mid to long term may have no treatment option other than total knee arthroplasty. Alternate treatment strategies such as ACI may lead to several re-interventions and dissatisfied patients but may postpone total knee arthrosplasty as possibly the only option for these patients and are therefore considered a good treatment for focal degenerative cartilage defects. However, long-term studies are needed to evaluate whether cell-based cartilage grafts prevent total joint replacement in osteoarthritis. After 6 months, there was no difference in the scores of these 2 patients with an endoprosthesis and the rest of the study group. After 12 months, these two patients with a 'graft failure' had not yet improved and revealed worse results compared with the other patients. After 12 months or after 48 months, neither of the 2 patients reached 55 points in the Lysholm score or 45 points in the IKDC score, indicating that there might be a threshold for long-term graft survival and successful tissue regeneration. Even if graft regeneration takes a long time (from 2 to 3 years after surgery), a continuous improvement should be detected 6 to 12 months after surgery. In this context, the lack of clinical improvement, combined with insufficient MRI results, may be signs of long-term graft failure. In general, re-operations and graft failure after implantation of chondrocytes in a first-generation ACI procedure are caused by problems associated with the periosteal flap [12,18,57]. This inherent technical disadvantage of the original ACI procedure does not occur when using second-generation ACI grafts like BioSeed^®^-C that are void of any cover materials. In addition, the easy and secure fixation of BioSeed^®^-C along with the lack of any covering may reduce the operating time and may result in a less invasive procedure since there is no need to harvest periosteum from the tibia. Limitations of the study are the small number of patients and the lack of a control group. Since this observational case report study was initiated first to gain insights in the safety and effectiveness of treating consecutive cartilage defects with BioSeed^®^-C, the study is further limited by the lack of predefined primary outcome goals. The study was not performed in a randomized controlled manner in comparison with an appropriate control group or with an alternate treatment option like microfracturing. Furthermore, in young patients, degeneration of cartilage can be observed after trauma whereas well-defined osteoarthritis occurs predominantly in older patients. Therefore, first- or second-generation ACI will not be recommended unrestrictedly for the treatment of focal osteoarthritic cartilage defects. However, this is the first study showing long-term results using the second-generation ACI graft BioSeed^®^-C in patients with osteoarthritic and/or degenerative changes of the knee. The study presents a continuous objective patient evaluation including clinical scores and control MRI over the course of a period of 4 years.

## Conclusions

This extensive case report shows promising results after implantation of the second-generation autologous cartilage graft, BioSeed^®^-C, for the treatment of focal degenerative cartilage defects of the knee. Clinical evaluation 4 years after implantation showed that the treatment of focal osteoarthritic defects with BioSeed^®^-C leads to significant improvement of the patients' condition as documented by reliable clinical outcome scores and by cartilage regeneration as well as articular resurfacing as assessed by MRI. The good clinical results found 1 year after implantation of BioSeed^®^-C lasted and remained stable for at least 4 years. Nevertheless, further long-term studies are needed to evaluate whether cell-based cartilage grafts prevent total joint replacement in osteoarthritis.

## Abbreviations

ACI: autologous chondrocyte implantation; ANOVA: analysis of variance; ICRS: International Cartilage Repair Society; IKDC: International Knee Documentation Committee; KOOS: Knee injury and Osteoarthritis Outcome Score; MRI: magnetic resonance imaging.

## Competing interests

CK is an employee of TransTissue Technologies GmbH, which is a subsidiary of BioTissue Technologies GmbH (Freiburg, Germany). BioTissue Technologies GmbH produces and distributes BioSeed^®^-C. CE works as a consultant for BioTissue Technologies GmbH. All other authors declare that they have no competing interests.

## Authors' contributions

PCK helped to carry out the data evaluation, to draft the manuscript, and to perform the patient data collection and participated in data evaluation. CK helped to carry out the data evaluation and to draft the manuscript. SM and CO helped to perform the patient data collection and participated in data evaluation. CE conceived the study, participated in its design and coordination, performed the surgical procedures, and was involved in the patient data collection and interpretation. All authors read and approved the final manuscript.
